# Docosahexaenoic acid (DHA) promotes immunogenic apoptosis in human multiple myeloma cells, induces autophagy and inhibits STAT3 in both tumor and dendritic cells

**DOI:** 10.18632/genesandcancer.131

**Published:** 2017-01

**Authors:** Donatella D’Eliseo, Livia Di Renzo, Angela Santoni, Francesca Velotti

**Affiliations:** ^1^ Department of Molecular Medicine, Pasteur Italia Laboratory, Rome, Italy; ^2^ Department of Experimental Medicine, Istituto Pasteur-Fondazione Cenci Bolognetti, Sapienza University of Rome, Rome; ^3^ IRCCS Neuromed, Pozzilli, IS, Italy; ^4^ Department of Ecological and Biological Sciences (DEB), La Tuscia University, Largo dell’Università, Viterbo, Italy

**Keywords:** docosahexaenoic acid-DHA, immunogenic cell death, autophagy, STAT3, dendritic cells-DCs

## Abstract

Docosahexaenoic acid (DHA), a ω-3 polyunsaturated fatty acid found in fish oil, is a multi-target agent and exerts anti-inflammatory and anticancer activities alone or in combination with chemotherapies. Combinatorial anticancer therapies, which induce immunogenic apoptosis, autophagy and STAT3 inhibition have been proposed for long-term therapeutic success. Here, we found that DHA promoted immunogenic apoptosis in multiple myeloma (MM) cells, with no toxicity on PBMCs and DCs. Immunogenic apoptosis was shown by the emission of specific DAMPs (CRT, HSP90, HMGB1) by apoptotic MM cells and the activation of their pro-apoptotic autophagy. Moreover, immunogenic apoptosis was directly shown by the activation of DCs by DHA-induced apoptotic MM cells. Furthermore, we provided the first evidence that DHA activated autophagy in PBMCs and DCs, thus potentially acting as immune stimulator and enhancing processing and presentation of tumor antigens by DCs. Finally, we found that DHA inhibited STAT3 in MM cells. STAT3 pathway, essential for MM survival, contributed to cancer cell apoptosis by DHA. We also found that DHA inhibited STAT3 in blood immune cells and counteracted STAT3 activation by tumor cell-released factors in PBMCs and DCs, suggesting the potential enhancement of the anti-tumor function of multiple immune cells and, in particular, that of DCs.

## INTRODUCTION

Docosahexaenoic acid (DHA; 22:6) is a long chain ω-3 polyunsaturated fatty acid (PUFA) primarily found in fish oil, that has been shown to have many health benefits in chronic diseases, such as inflammation-mediated diseases and cancer [1, 2]. Indeed, DHA can exert anti-cancer activity towards several established solid and hematologic tumors [3–5]. In addition, DHA has been proposed as a non-toxic adjuvant to improve efficacy of conventional cancer therapies, since it can enhance the antitumor activity of chemotherapeutics, especially towards drug resistant cells, with no adverse effects [6–9]. The mechanisms underlying the anti-neoplastic effects of DHA are still unclear and need to be elucidated. Accumulating evidence indicates that DHA exhibits multiple mechanisms of action, including the in vitro and in vivo down-modulation of cancer cell proliferation and survival, invasiveness and metastasis, angiogenesis and inflammation [2, 5, 10–15]. It has been well documented that DHA represents a multi-target anticancer agent, since cell membrane enrichment with DHA induces changes in the distribution and function of several molecules, including key signaling mediators of cell survival and death [2, 5]. All these considerations greatly support investigations to further analyze and clarify the anti-cancer effects of DHA, to assess its potential use in cancer therapy either alone [2–5] or in combinatorial strategies [6–9] to improve the efficacy and tolerability of conventional anticancer treatments.

Multiple Myeloma (MM) is a haematologic cancer of plasma cells infiltrating the bone marrow, where cancer cells, influenced by the microenvironment, become resistant to most drugs and apoptotic signals. MM cells are characterized by the release of high levels of cytokines, which maintain cell autonomous proliferation/survival as well as suppress the immune response [16–18]. Indeed, the growth of MM, as of other tumors, is mainly due to the effect of tumor-released factors and relays on the constitutive activation of several pro-survival pathways including STAT3 [16, 19]. STAT3 constitutive activation in MM cells also confers them drug resistance [19–21]. In addition, STAT3, beyond its oncogenic role at the tumor cell level, has potent immunosuppressive effects in the tumor microenvironment, affecting the function of multiple lymphoid and myeloid cell types including dendritic cells (DCs) [22]. Indeed, in MM patients, tumor-released suppressive factors (such as TGF-*β*, IL-10, IL-6 and VEGF) can abrogate DC function, by activation of STAT3 [23–25].

DCs are at the center of the immune system owing to their ability to induce tumor-specific effector T cells, that can reduce the tumor mass and induce immunological memory to control tumor relapse, thus leading to long-term survival [26]. Therefore, DCs represent an essential target in efforts to generate therapeutic immunity against cancer, especially during chemotherapy [26, 27]. The capability to stimulate protective anticancer immune responses by DCs depends on multiple conditions and multiple strategies have been proposed. One of the strategies is the so-called immunogenic chemotherapy, based on the capability of some chemotherapeutic agents to promote an immunogenic cancer cell apoptosis [27–29]. This means that a chemotherapeutic agent simultaneously induces cancer cell apoptosis and autophagy, where dying cancer cells emit a spatiotemporal-defined combination of specific damage-associated-molecular-patterns (DAMPs), that, loaded together with multiple tumor antigens in intact autophagosomes, are taken up by DCs, inducing their maturation, activation and antigen cross-presentation, thus leading to the stimulation of an effective anti-tumor T cell response [27–29]. In addition, new studies have proposed that, together with immunogenic chemotherapeutics, autophagy enhancers should expand the pharmacological arsenal and augment the efficacy of cancer immunotherapy [30]. Indeed, autophagy enhancers affect cancer by attenuating tumor-promoting inflammation and stimulating antitumor immunity [30]. In particular, autophagy in DCs increases the processing and presentation of tumor antigens by both MHC class II and I molecules, thereby stimulating anti-tumor T cell response [30]. Finally, for all the reasons mentioned before, another promising therapeutic strategy for MM, and for other cancers as well, consists in STAT3 targeting [21, 31]. In fact, pharmacologic inhibitors of STAT3 pathway on the one hand affect cancer cell survival, suppressing tumor cell autonomous tumorigenesis [20, 21, 31] and on the other hand inhibit STAT3 inflammatory signaling in the hematopoietic system, eliciting multicomponent antitumor immune responses including those mediated by DCs [32–34]. Indeed, STAT3 depletion in DCs improves cancer immunotherapy, by enhancing their ability to induce tumor antigen-specific T cells and promoting their resistance to cancer cell-derived inhibitory factors [35]. To notice, STAT3 inhibitors on tumor cells, used in combinatorial therapy with immunogenic chemotherapeutics such as anthracyclines, improve the outcome of immunogenic chemotherapy by stimulating the type-1 interferon production by cancer cells [36].

In this study, we investigated the promotion of cell death, the activation of autophagy and the inhibition of STAT3 by DHA in MM cells as well as in peripheral blood mononuclear cells (PBMCs) and DCs. In particular, we explored whether DHA promoted immunogenic apoptosis in MM cells, first analyzing its capability to trigger the emission of specific DAMPs by apoptotic cancer cells; then examining its capability to enhance autophagy in MM cells and the role of autophagy in cell viability. Lastly, we directly verified the immunogenicity of cell death induced by DHA by investigating whether MM cells undergoing DHA-mediated apoptosis were capable of activating DCs; we compared this effect to that obtained by using lipopolysaccharide (LPS), the classical DC activator. We also investigated the capability of DHA to activate autophagy in immune cells, such as PBMCs and DCs. Finally, we examined whether DHA was capable to inhibit STAT3 activation in MM cells, PBMCs and DCs. On this last point, we evaluated the capability of DHA to counteract STAT3 activation triggered by tumor cell-released factors in both PBMCs and DCs.

## RESULTS

### DHA induces apoptosis in MM cells with no cytotoxic effects on PBMCs

**Figure 1:**
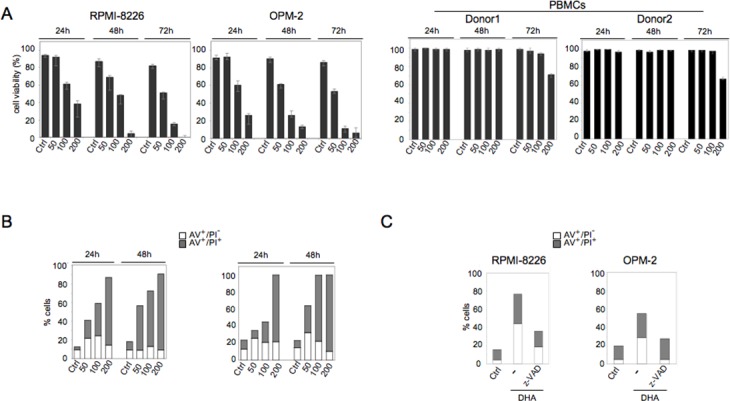
DHA induces apoptosis in MM cells and does not affect PBMC viability A. DHA decreases viability of MM cell lines in a dose- and time-dependent manner, whereas it does not affect the survival of PBMCs derived from healthy donors. RPMI-8226, OPM-2 and PBMCs were cultured with vehicle (Ctrl) or DHA (μM) and their viability evaluated by trypan blue exclusion assay; mean of the percentage of cell surviaval plus SD of three independent experiments is indicated; B. RPMI-8226 and OPM-2 were cultured with vehicle (Ctrl) or DHA (μM) and apoptosis was assessed by Annexin V-FITC (AV) and propidium iodide (PI) cell staining and flow cytofluorimetry; representative experiments out of three; C. RPMI-8226 and OPM-2 were cultured with vehicle (Ctrl) or 100 μM DHA for 24 hours in the absence or presence of z-VAD-FMK (50 μM) and analyzed for apoptosis by AV and PI cell staining; representative experiments out of three.

To investigate the promotion of cell death by DHA in human MM cells, we first analyzed the induction of cytotoxicity by DHA in MM cells *vs* normal PBMCs. To this purpose, two MM cell lines, RPMI-8226 and OPM-2, as well as PBMCs from two healthy donors were cultured in the presence of increasing doses of DHA (50-200 μM) for different time periods (24, 48 and 72 hours) and the effect of DHA on cell viability was determined by the trypan-blue exclusion assay. As shown in Figure 1A, DHA treatment resulted in a dose- and time-dependent cytotoxicity in both MM cell lines, whereas it did not affect the viability of normal PBMCs.

To characterize the cell death induced by DHA in MM cells, we examined the occurrence of apoptosis by immunofluorescence, using the phosphatidylserine (PS)-binding annexin V (AV) and the vital dye propidium iodide (PI), in RPMI-8226 and OPM-2 cells cultured in the presence of increasing doses of DHA (50-200 μM) for 24 and 48 hours. As shown in Figure 1B, apoptotic cell death occurred in both MM cell lines and took place in a dose- and time-dependent manner. To confirm tumor cell death by apoptosis, MM cells were treated with 100 μM DHA for 24 hours in the presence or in the absence of z-VAD pan-caspase inhibitor. As shown in Figure 1C, z-VAD inhibited apoptosis mediated by DHA in both cell lines. These results showed that DHA induced apoptotic cell death in MM cells, whereas it did not affect the viability of normal PBMCs.

### DHA promotes immunogenic apoptosis in MM cells

**Figure 2:**
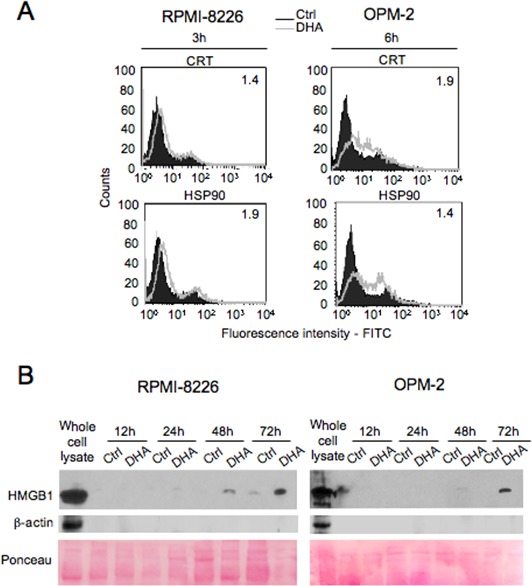
DHA triggers the emission of immunogenic DAMPs by MM cells A. RPMI-8226 and OPM-2 were cultured with vehicle (Ctrl) or 100 μM DHA for 3 and 6 hours, respectively; cell surface immunofluorescence staining using anti-CRT, anti-HSP90 or isotype control antibodies was analyzed by flow cytofluorimetry, while gating on the viable population and excluding dead cells stained with PI; numbers indicate the ratio of the mean fluorescence intensity (MFI) of DHA treated cells/MFI of control cells. B. RPMI-8226 and OPM-2 were cultured with vehicle (Ctrl) or 100 μM DHA for the indicated times; then, tumor cell conditioned media were collected and the presence of HMGB1 was analyzed by Western blot; β-actin was used as intracellular protein control and Ponceau staining as loading control. Representative experiments out of three.

Apoptosis can be immunogenic or tolerogenic, depending on its ability to trigger the emission by apoptotic cancer cells of a spatiotemporally-defined combination of DAMPs, which are able to stimulate antitumor immune responses through antigen presenting cells (APCs) such as DCs [27, 28, 37, 38]. Distinctive features of immunogenic apoptosis include the cell surface exposure of calreticulin (CRT) [39] and/or HSP90 [40] in pre- or early-apoptotic stages, as well as the release of non-histone chromatin protein high mobility group box 1 (HMGB1) by cancer cells in late-apoptosis or secondary necrosis [41]. Therefore, we investigated whether DHA-mediated apoptosis in MM cells had the ability to trigger the emission of the specific DAMPs in the proper spatiotemporally-defined combination. We found that both CRT and HSP90 were exposed on the cell surface of RPMI-8226 and OPM-2 cells treated with DHA for 3 and 6 hours, respectively (Figure 2A). Moreover, HMGB1 was released in the conditioned medium by both RPMI-8226 (left panel) and OPM-2 (right panel) cells at late apoptotic stages (Figure 2B). All together, these results suggested that apoptosis mediated by DHA in MM cells was immunogenic.

### DHA activates autophagy in MM cells, PBMCs and DCs

**Figure 3:**
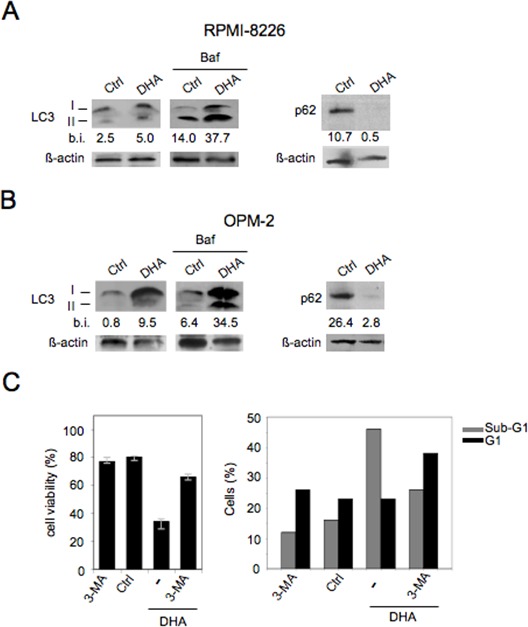
DHA enhances autophagy in MM cells, which contributes to DHA-induced cell death RPMI-8226 (A) and OPM-2 (B) were cultured with vehicle (Ctrl) or 100 μM DHA for 24 hours in the presence or in the absence of Bafilomycin (Baf) and the expression of the autophagic markers such as LC3I/II and p62 was analyzed by Western blot; β-actin was included as control; numbers indicate band intensities (b.i.) = band volume/area x mean pixel intensity, normalized for β-actin and quantified using Quantity One 1-D analysis software; C. RPMI-8226 cells were cultured for 24 hours with vehicle (Ctrl) or 100 μM DHA in presence or absence of 3-MA (0.3 mM) and their viability assessed by trypan blue exclusion assay (left panel) and cytofluorimetry cell cycle analysis of sub-G1 events, representing apoptotic cells (right panel). Representative experiments out of three.

Another required feature of immunogenic apoptosis includes the capability of chemotherapeutics to activate autophagy in cancer cells [29, 30]. Therefore, we explored the activation of autophagy in MM cells by DHA and its role in cancer cell viability. To this purpose, the main autophagic markers such as LC3I/II and p62 [42] were evaluated by Western blot analysis. As shown in Figure 3A-B, LC3II formation increased both in RPMI-8226 and in OPM-2 cells cultured with DHA (100 μM) for 24 hours and accumulated in the presence of Bafilomycin (Baf), an inhibitor of ATP vacuolase that, by blocking LC3II degradation, allows to evaluate LC3 formation and consequently the completeness of the autophagic flux [42]. Conversely, p62 decreased (Figure 3A-B), further indicating that DHA was able to activate a complete autophagy in MM cells. Next, the role of autophagy activated by DHA in MM cell viability was investigated by the administration of the autophagic inhibitor 3-methyladenine (3-MA). As shown in Figure 3C (left panel), the viability of RPMI-8226 cells was increased when 3-MA was applied. According to this observation, we also found that 3-MA partially decreased the percentage sub-G1 events, indicative of apoptotic nuclei, while increased the percentage of cells in the G1 phase (Figure 3C, right panel). These results implied that autophagy by DHA played a pro-apoptotic role in MM cells and that contributed to apoptotic cell death mediated by DHA.

**Figure 4:**
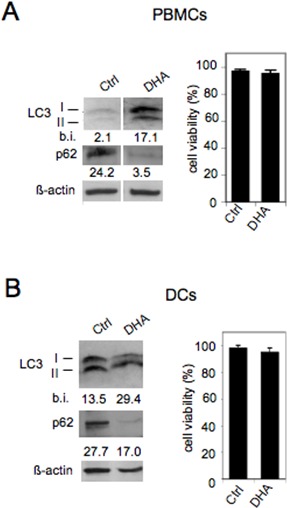
DHA enhances autophagy in PBMCs and DCs PBMCs (A) and DCs (B) derived from healthy donors were cultured with vehicle (Ctrl) or 100 μM DHA for 24 hours and the expression of the autophagic markers LC3I/II and p62 was analyzed by Western blot; β-actin was included as control; numbers indicate band intensities (b.i.) = band volume/area x mean pixel intensity, normalized for β-actin and quantified using Quantity One 1-D analysis software; the viability of PBMCs (A) and DCs (B) was assessed by trypan blue exclusion assay. Representative experiment out of three.

Then, we investigated the activation of autophagy by DHA in immune cells. As shown in Figure 4, increased LC3II formation and decreased p62 appeared in both PBMCs (panel A) and DCs (panel B) following their treatment with 100 μM DHA for 24 hours. Moreover, it is worth noting that, according to the results shown in Figure 1A, DHA did not induce toxic effects in either PBMCs (panel A) or DCs (panel B) (Figure 4). These findings indicate that DHA is an enhancer of autophagy in immune cells as well, potentially decreasing their inflammatory activity and enhancing their immune response against tumor antigens [30].

### DHA-triggered immunogenic apoptosis in MM cells activates DCs

**Figure 5:**
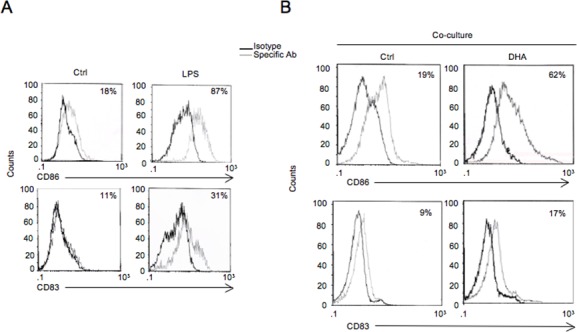
DHA-triggered immunogenic apoptosis in MM cells activates DCs Immature DCs (iDCs), generated from PBMC-derived CD14+ monocytes cultured with GM-CSF and IL-4 for 6 days, were co-cultured with vehicle- (Ctrl) or DHA-treated RPMI-8226 cells for 24 hours and the expression of DC differentiation (CD83) and activation (CD86) markers was analyzed by immunofluorescence and flow cytometry (B). As positive control of DC activation, cells were treated with LPS (100 ng/ml) for the same time (A). Representative experiment out of three.

Since all the present results indicated that DHA-mediated apoptosis in MM cells had the features of immunogenic apoptosis, we investigated whether cancer cells undergoing apoptosis by DHA were able to activate DCs. To this purpose, immature DCs (iDCs), generated from human peripheral blood derived CD14+ monocytes cultured with human recombinant granulocyte-macrophage colony stimulating factor (GM-CSF) and interleukin-4 (IL-4) for 6 days, were co-cultured with DHA-induced apoptotic RPMI-8226 cells for 24 hours and the expression of DC activation markers was analyzed. As positive control of DC activation, iDCs were treated with LPS (100 ng/ml) for the same time (Figure 5A). As shown in Figure 5B, DHA up-regulated the expression of DC differentiation and activation markers CD83 and CD86, respectively, as evidenced by flow cytometric analysis.

### DHA inhibits STAT3 activation in MM cells, PBMCs and DCs

**Figure 6:**
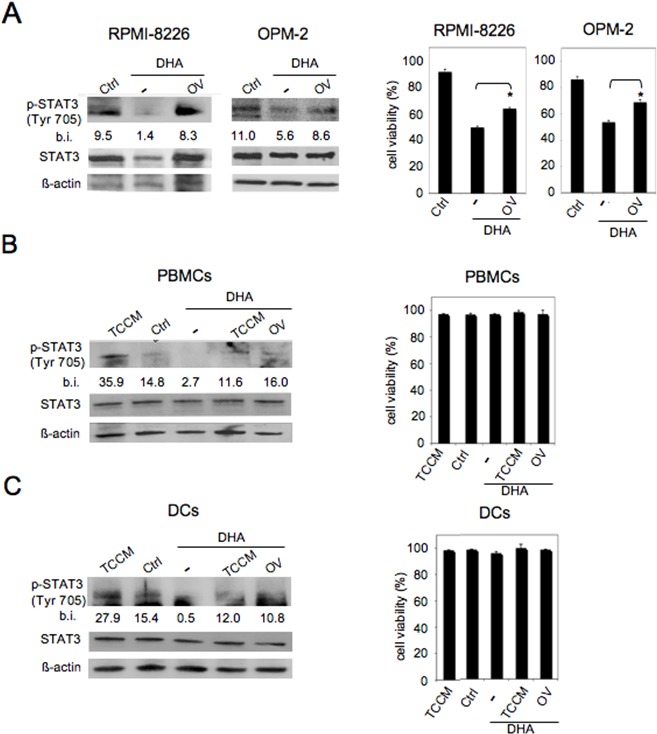
DHA inhibits STAT3 pathway in MM cells, PBMCs and DCs A. RPMI-8226 and OPM-2 were cultured with vehicle (Ctrl) or 100 μM DHA for 24 hours in the presence or absence of sodium orthovanadate (OV) (150 μM) and STAT3 tyrosine phosphorylation (p-STAT3) was evaluated by Western blot (left panels); total STAT3 and β-actin were included as control; numbers indicate band intensities (b.i.) = band volume/area x mean pixel intensity, normalized for β-actin and quantified using Quantity One 1-D analysis software; viability of MM cells was assessed by trypan blue exclusion assay (right panels); *p< 0.000; PBMCs from healthy donors (B) and iDCs (C), generated from PBMC-derived CD14+ monocytes cultured with GM-CSF and IL-4 for 6 days, were cultured with vehicle (Ctrl) or 100 μM DHA for 24 hours in the presence or absence of tumor cell conditioned medium (TCCM) or OV (150 μM), and evaluated for STAT3 tyrosine phosphorylation by Western blot (left panels); total STAT3 and β-actin were included as control; numbers indicate band intensities (b.i.) = band volume/area x mean pixel intensity, normalized for β-actin and quantified using Quantity One 1-D analysis software; the viability of PBMCs and DCs was assessed by trypan blue exclusion assay (right panels). Representative experiments out of three.

The inhibition of the STAT3 pathway in both cancer and immune cells (particularly myeloid populations) constitutes an important target for cancer therapy, including MM therapy [19–22, 24, 25, 31–35]. Therefore, we investigated whether DHA was capable to inhibit STAT3 in MM cells as well as in PBMCs and DCs. To this purpose, both RPMI-8226 and OPM-2 cells were treated with 100 μM DHA for 24 hours and phosphorylated STAT3 (p-STAT3) was evaluated by Western blot analysis. As shown in Figure 6A left panels, DHA strongly suppressed STAT3 tyrosine phosphorylation in both MM cell lines. Moreover, DHA-induced STAT3 de-phosphorylation was reduced by the broad-acting phosphatase inhibitor sodium orthovanadate (OV) [43] (Figure 6A, left panels), suggesting that tyrosine phosphatases were involved in DHA-mediated STAT3 de-phosphorylation. Then, we investigated the possible role of STAT3 in DHA-mediated cytotoxicity in MM cells. As shown in Figure 6A, right panels, we found that OV treatment reduced the cytotoxicity mediated by DHA both in RPMI-8226 and OPM-2 cells, suggesting that STAT3 inhibition was involved in DHA-mediated cell death.

Next, to investigate the inhibition of the STAT3 pathway in immune cells, PBMCs and DCs derived from healthy donors were treated with 100 μM DHA for 24 hours in the absence or presence of MM cell-derived conditioned medium and STAT3 phosphorylation was evaluated by Western blot analysis. As shown in Figure 6B-C (left panels), DHA strongly suppressed both constitutive and tumor cell conditioned medium (TCCM)-induced activation of STAT3 in both PBMCs and DCs, indicating that DHA can inhibit STAT3 signaling in immune cells and has the potential to counteract STAT3 activation induced by MM cell-released factors. Finally, according to the results obtained in tumor cells, DHA-induced STAT3 de-phosphorylation was reduced by OV (Figure 6B-C, left panels), suggesting a role for tyrosine phosphatases in DHA-mediated STAT3 de-phosphorylation in immune cells. Moreover, according to previous results (Figures 1A and 4), DHA treatment did not affect the viability of PBMCs and DCs (Figure 6 B-C, right panels).

## DISCUSSION

Despite progress made in recent years in cancer chemotherapy, this therapeutic strategy alone has not provided satisfactory clinical results in terms of the long-term survival of patients, mainly related to the development of drug resistance by cancer cells, toxicity towards normal cells and impaired immunity [27, 44]. Evidence exists that the host immune system plays a major role in long-term therapeutic success and combinatorial multi-targeted strategies, where chemotherapeutic agents are combined with immunotherapies, are needed to completely eradicate cancer diseases [27, 44].

DHA represents one of the most promising natural products in the therapy of various human inflammation-mediated diseases and cancer, being able to target multiple key molecules in different compartments of tumor and normal cells [1–5, 14, 15]. Moreover, the well documented capability of DHA to induce selective cytotoxicity against several types of solid and hematologic cancer cells in vitro and in vivo without exerting toxic effects in the corresponding normal cell types [2, 5, 45–49] makes DHA a potentially ideal anticancer agent.

In this study, we found that DHA induced immunogenic apoptosis in MM cells, while, according to the literature [5, 45], did not induce cytotoxicity in normal PBMCs and DCs. The immunogenicity of cell death induced by DHA in MM cells was first indicated by the finding that MM apoptosis was associated with the correct spatiotemporally-defined cell surface exposure not only of CRT, as we showed in an earlier study on other tumor models [50], but also of HSP90, followed by the extra-cellular release of HMGB1, all specific DAMPs representing distinctive features of immunogenic apoptosis [27, 28, 38]. Then, we showed that DHA activated autophagy in MM cells. This is important, since autophagy, although dispensable for chemotherapy-induced cell death, is required for its immunogenicity, as it enhances the release of specific DAMPs (including ATP, HMGB1, uric acid), allowing cancer cells to attract DCs and T lymphocytes into the tumor bed [27, 30]. Noteworthy, autophagy in cancer cells can also indirectly promote “cross-presentation” of tumor antigens by facilitating antigen release from dying cells, thereby increasing extracellular antigen availability [30]. It has been recently shown that cancers, in which autophagy was upregulated, exhibit higher density of CD8+ T cells and lower number of Foxp3+ T regulatory cells (Treg) in the tumor bed [30, 51]. Moreover, we found that DHA-activated autophagy in MM cells amplified their apoptotic cell death, since the inhibition of autophagy by 3-MA increased cancer cell viability. Our findings are consistent with earlier studies demonstrating that DHA can simultaneously promote apoptosis and autophagy in different solid tumors in vitro and in vivo [52, 53]. Although the mechanism underlying DHA-activated autophagy has not yet been fully elucidated, it has been proposed to be dependent on the inhibition of mTOR (a negative regulator of autophagy initiation) by DHA, via AMPK activation and PI3K/Akt inhibition [53]. Finally, we directly showed the immunogenicity of DHA-mediated apoptosis by the capability of apoptotic MM cells to activate DCs.

Next, we provided the first evidence that DHA was capable to activate autophagy in PBMCs and DCs, while did not affect their viability. This is an important point, since, in tumor bearing mice or cancer patients, tumor-infiltrating APCs are often functionally compromised and APC autophagy needs to be stimulated to facilitate processing and cross-presentation of tumor antigens by MHC molecules, ensuring the generation of effective antitumor T cells [30]. Currently, autophagy is considered as an immune stimulator, which tunes down inflammation and boosts adaptive immunity against tumor progression [30].

Finally, we showed that DHA targeted STAT3 signaling by strongly inhibiting STAT3 phosphorylation in tumor cells as well as in PBMCs and DCs. STAT3 inhibition in cancer cells appeared to be involved in the apoptotic process promoted by DHA, as STAT3 de-phosphorylation was associated with cancer cell death and treatment with a phosphatase inhibitor inhibited the cancer cell death. Our findings are consistent with previous data, showing the capability of DHA to inhibit STAT3 in other tumor cell models [54, 55]. Moreover, new data have shown that STAT3 inhibitors, in combinatorial therapy with anthracycline-based immunogenic chemotherapy, potentiated the immunogenic chemotherapy [32]. Noteworthy, the capability of DHA alone both to promote immunogenic apoptosis and to inhibit STAT3 in MM cells, makes this agent potentially more advantageous than conventional immunogenic chemotherapeutics. In addition, we found that DHA inhibited STAT3 in peripheral blood immune cells and, more importantly, has the potential to counteract STAT3 activation by tumor cell-released factors in PBMCs and DCs, with no toxic effects. Although STAT3 inhibitory activity by DHA has been previously reported in hepatocytes [56] and adipocytes [57] as a mechanism contributing to the anti-inflammatory effect of DHA, the capability by DHA to induce STAT3 inhibition in immune cells, including DCs, has not been described before. Our findings, showing STAT3 inhibition in immune cells by DHA, might result in an enhancement of the antitumor response of multiple peripheral blood immune cell populations, especially that of DCs [22–25]. To the best of our knowledge, no or little studies have investigated the effect of DHA-based clinical trials in cancer patients on the anti-tumor immune response. Eltweri et al. [15], reviewing ω3-PUFAs-based trials in the pre- and/or post-operative setting in gastrointestinal cancers, reported in several studies the reduction of inflammatory markers and the improvement of the immune function, such as an increased resistance to infectious diseases.

In conclusion, our findings, indicating that DHA promotes immunogenic apoptosis in MM cells with no toxicity on PBMCs and DCs, activates autophagy and inhibits STAT3 in MM cells as well as in PBMCs and DCs, strongly encourage the potential use of this multi-target agent in cancer therapy either alone or in combinatorial strategies, to potentiate conventional immunogenic or non-immunogenic chemotherapies.

## MATERIALS AND METHODS

### Tumor Cells

The human MM cell lines RPMI-8226 and OPM-2 were provided by P. Trivedi (“ La Sapienza” University of Rome, Italy). Cell lines were maintained at 37°C and 5% CO2 in RPMI 1640 (Sigma Aldrich) supplemented with 10% FCS (Hyclone), 100 mg/ml streptomycin and 100 IU/ml penicillin (EuroClone). All cell lines were mycoplasma free (EZ-PCR Mycoplasma test kit; Biological Industries).

### Peripheral Blood Mononuclear Cells (PBMCs) and Immature DC (iDC) generation

Human PBMCs from healthy donors were isolated by Lymphoprep gradient centrifugation (Nycomed). Monocytes were isolated by immunomagnetic cell separation using anti-CD14-conjugated microbeads (Miltenyi Biotec, 1300-50-201). To induce the differentiation of iDCs, monocytes were cultured for 6 days with human recombinant granulocyte-macrophage colony stimulating factor (GM-CSF) (50 ng/mL) (Milteny Biotec, 130-093-865) and interleukin-4 (IL-4) (20 ng/mL) (Miltenyi Biotec, 130-095-373), as previously described [58].

### iDC/tumor cell co-cultures

iDCs were co-cultured with DHA-treated tumor cells for 24 hours, at a 1:2 iDC/tumor cell ratio, as previously described [58].

### Drug treatment

Cells were cultured with DHA (Sigma-Aldrich, D2534-100MG), dissolved in ethanol solution or with ethanol solution alone (Ctrl) at the indicated doses, for the indicated times. In some experiments, z-VAD-FMK pan-caspase inihibitor (50 μM) (Calciochem; 219011) or sodium orthovanadate (OV) (150 μM) (Sigma Aldrich, 450243) were added to the cell culture 30 minutes before DHA (100 μM) treatment for 24 hours; in others, 3-methyladenine (3-MA) (0.3 mM) (Santa Cruz Biotechnology Inc., sc-205596) was added 6 hours after DHA (100 μM) treatment for 24 hours. For the autophagic investigation, MM cells were cultured with DHA (100 μM) for 24 hours and then treated with Bafilomycin A1 (Baf) (20 nM) (Santa Cruz Biotechnology Inc., sc-201550), an inhibitor of vacuolar-H+-ATPase, for the last 2 hours. For DC activation, iDCs were cultured with LPS (100 ng/mL) for 24 hours.

### Cell viability and Apoptosis assay

After each chemical treatment, cell viability was assessed by the trypan blue dye exclusion assay. Live cells were counted by light microscopy using a Neubauer hemocytometer. Apoptosis was assessed by annexin V-FITC and propidium iodide staining, as previously described [59]. DNA fragmentation was quantified by flow cytometry of hypodiploic (sub-G1) DNA after cell fixation and PI staining, as previously described [60].

### Immunofluorescence and flow cytometric analysis

Immunofluorescence was performed using antibodies against CRT (FMC75; MBL International SR601D MBL), HSP90 (AC88; StressGen ADI-SPA-830-D), CD14 (Miltenyi Biotec, 130-080-701), CD1a (BD Biosciences Pharmingen, 555807), CD86 (Milteny Biotec, 130-094-878), CD83 (Miltenyi Biotec, 130-094-181) or appropriate isotype control antibodies. Samples were analyzed by a FACSCalibur (Becton Dickinson) or EPICS XL (Beckman Coulter) flow cytometer. DCs were gated according to their FSC and SSC properties. At least 5 × 10^3^ events were acquired for each sample.

### Western blot analysis

Cell lysates were prepared by a solution containing 50 mM TRIS-HCl pH 7.6, 150 mM NaCl, 0.5% TRITON X-100, 0.5% Sodium deoxycolate, 0.1% SDS and the protease inhibitor mixture “Complete” (Roche Diagnostic GmbH). Proteins were separated by SDS-PAGE, blotted onto nitrocellulose (Whatman-Protan BA85) and incubated with appropriated primary antibodies specific for: STAT3 (BD Transduction Laboratories; 610189), phosphoSTAT3 (pY705) (BD Transduction Laboratories; 612356), LC3 (Novus Biologicals, NB100-2220SS), p62 (BD Transduction Laboratories, 610832), HMGB1 (Abcam, 18256) or β-actin Ac-40 (Sigma-Aldrich, A4700). The reaction was revealed by horseradish peroxidase (HRP)-coupled secondary reagents (Bio-Rad) and developed by enhanced chemiluminescence (Amersham ECL Western Blotting Detection Reagent). Band intensities (b.i.) = band volume/area x mean pixel intensity, normalized for β-actin, were quantified using Quantity One 1-D analysis software (Bio-Rad).

### Statistics

Student’s *t* test was used for all analyses; *p* < 0.05 was considered significant. All experiments were performed at least three times.

## ABBREVIATIONS

DHA: docosahexaenoic acid
ω-3 PUFA: ω-3 polyunsaturated fatty acid
MM: multiple myeloma
DCs: dendritic cells
PBMCs: peripheral blood mononuclear cells
DAMPs: damage associated molecular patterns
CRT: calreticulin
HMGB1: protein high mobility group box 1
GM-CSF: granulocyte-macrophage-colony stimulating factor
IL-4: interleukin-4
LC3: microtubule-associated protein 1 light chain 3
3-MA: 3-methyladenine
OV: sodium orthovanadate
LPS: lipopolysaccharide
TCCM: tumor cell conditioned medium
